# Unusual Presentation of a Granulocytic Sarcoma

**DOI:** 10.7759/cureus.53980

**Published:** 2024-02-10

**Authors:** Daniel Swink, Laura Mena Albors, Danis Lester, Carrie D Watson

**Affiliations:** 1 General Surgery, University of Central Florida College of Medicine, Ocala, USA

**Keywords:** colorectal cancer surgery, metastatic granulocytic sarcoma, colon resection, colon mass, surgery general, granulocytic sarcoma

## Abstract

A granulocytic sarcoma is an unusual tumor outside of bone marrow. It is composed of immature cells of the granulocytic cell line. We present a rare case of a 76-year-old male with a history of myelodysplastic syndrome who presented with a large bowel obstruction secondary to lesions at the cecum and transverse colon. He underwent exploratory laparotomy with extended right hemicolectomy. A pathological examination confirmed a granulocytic sarcoma as the cause of the obstruction.

## Introduction

A granulocytic sarcoma (GS), also known as an extramedullary myeloid tumor or chloroma, is defined as an extramedullary tumor composed of immature cells of the granulocytic series. It rarely occurs in patients with acute myelogenous leukemia (AML), myelodysplastic syndrome, or chronic myelogenous leukemia (CML). GSs are usually located in the skin, lymph nodes, soft tissues, bone, and periosteum [1]. Other sites involved are the testes, kidneys, orbits, uterus, bladder, gingiva, and stomach [2]. Gastrointestinal GSs make up 7-15% of all GSs, the ileum being the most common site [1,3,4]. They are rarely located in the colon or rectum; less than 15 such cases have been reported. 

## Case presentation

**Figure 1:**
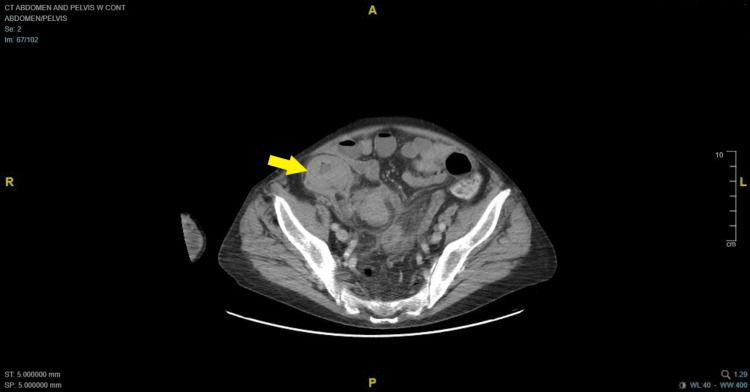
Thickening of the cecum

**Figure 2:**
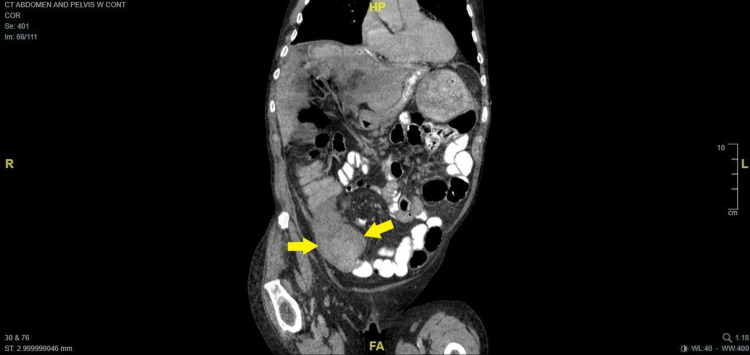
Thickening of the cecum

**Figure 3:**
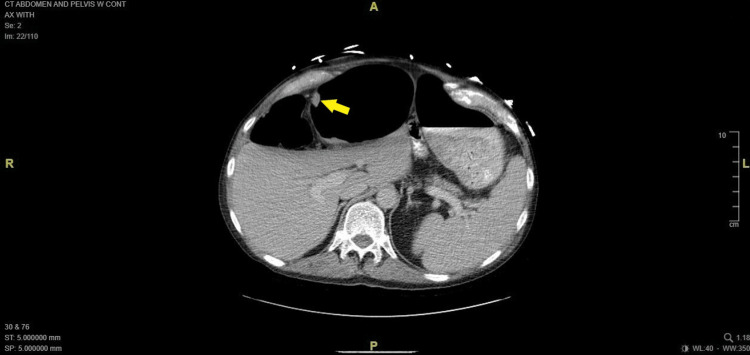
Intraluminal lesion of the transverse colon

A 76-year-old male with a history of myelodysplastic syndrome (MDS) presented with abdominal pain, nausea, vomiting, and diarrhea for 48 hours. He had been discharged from an outside hospital two weeks prior after he had been non-operatively managed for abdominal pain and blood per rectum. At the time of presentation to our facility, CT imaging demonstrated thickening of the right colon (Figures 1, 2) ) as well as intraluminal lesion at the transverse colon ( Figure 3).

His initial hemoglobin was 8.1 with a WBC count of 9.7 with mild distention and tenderness without peritoneal signs. The initial plan for management included a colonoscopy to evaluate the thickening of the cecum and rectal bleeding. On hospital day two, his WBC increased to 13.3; he had worsening abdominal pain with focal peritonitis. He was taken to the operating room for exploratory laparotomy.

**Figure 4:**
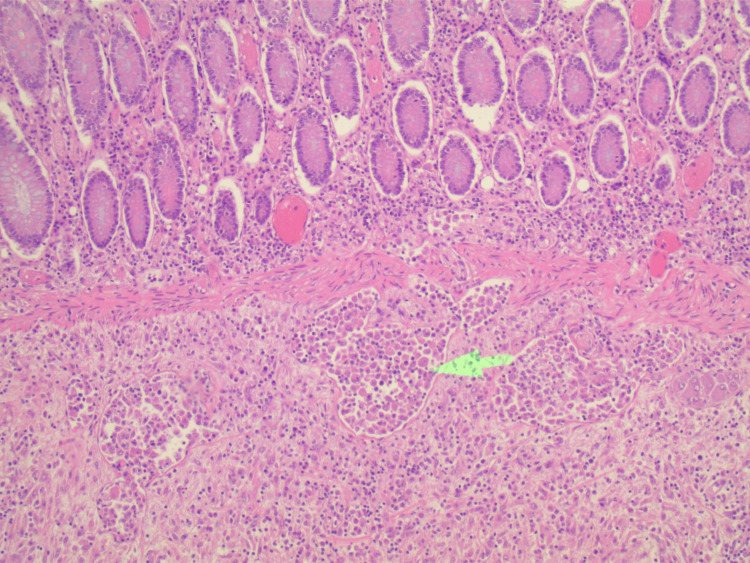
Colon with submucosal tumor identified by the green arrow

**Figure 5:**
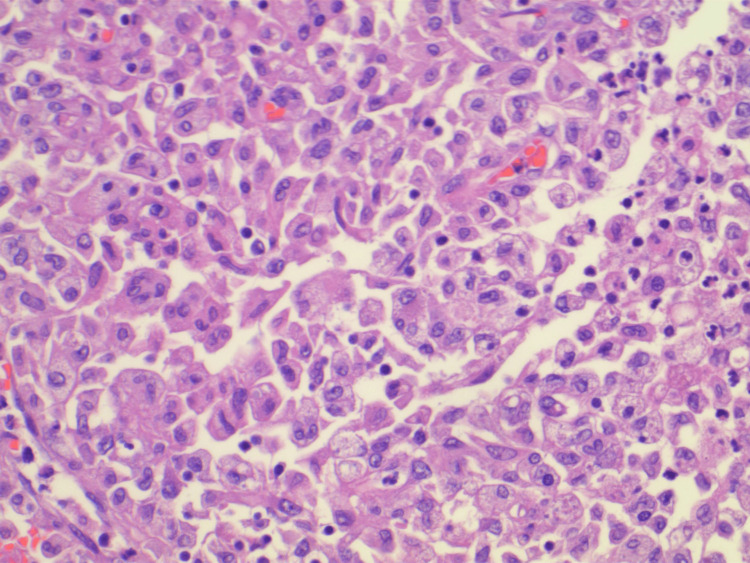
High-power field of the mesenteric tumor

A midline laparotomy incision was performed and the abdomen was explored. Two masses were identified, one in the cecum that was adherent to the pelvic side wall and a second distal to the hepatic flexure. The remainder of the colon was not involved and the small intestine had a segment that felt suspicious at the ileocolonic junction. The right and transverse colon was mobilized and the mass was found to be locally invading into the pelvic sidewall. The mass in the cecum was widely excised, while the mass in the transverse colon did not appear to be locally invasive. A margin of 5 cm distal to the transverse colon mass was marked and the colon specimen was taken. A 10 cm margin onto the terminal ileum was resected. An end ileostomy was created, as there was a concern about complications as intra-operatively the tumors did not appear to be of colonic origin. The pathology was consistent with extramedullary myeloid tumor at both the cecum and transverse colon (Figure 4). The mesenteric specimen was also positive for tumor cells, which can be seen in the high-power field (Figure 5).

The mass was perforated at the cecum and was adherent to the pelvic side wall. The mass was excised en bloc with negative margins at the cecum and transverse colon with 15/15 nodes involved. At the time of diagnosis, he had completed chemotherapy for myelodysplastic syndrome. The patient had an uncomplicated postoperative course. He tolerated the diet and was discharged home in stable condition. 

## Discussion

**Figure 6:**
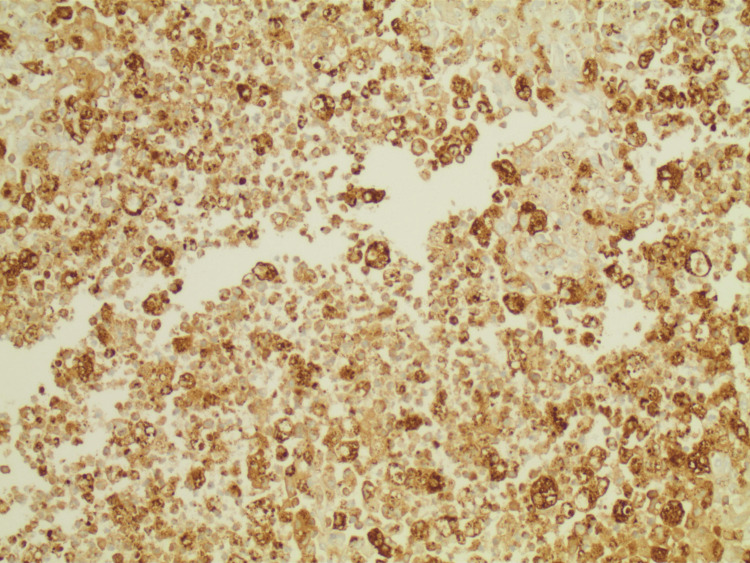
Myeloperoxidase stain of the tumor

Granulocytic sarcomas are rare entities estimated to affect 4-4.7% of patients with acute myelogenous leukemia [1]. In a patient with a history of MDS, they are concerning as potential heralds of transformation to AML. They are most commonly noted in the bone, lymph, peritoneum, and gastrointestinal (GI) tract [1,4]. Given the wide range of potential primary sites, presenting symptoms are variable, however are typically related to mass effect and bleeding. A GS is best localized through the use of MR or CT imaging, and tissue biopsy confirms the diagnosis [5]. The WHO recommends cytochemical stains of the tumor including chloroacetate, myeloperoxidase, and nonspecific esterase. A myeloperoxidase stain was obtained and was positive (Figure 6).

The treatment for GSs is chemotherapy; however, surgical intervention may be required in select cases. Given the rarity of the disease, no large studies have been conducted on prognosis [5]. We recommended having an index of suspicion for the development of these GSs in patients with a history of MDS; however, they should be treated as any other potential primary colon cancer. If not managed promptly, there is a risk of bowel obstruction and perforation, leading to increased mortality. A collected case series by Byrd et al. demonstrated poor outcomes in patients who had MDS accompanied by GS [6]. Treatments with systemic therapy have been associated with improved survival. 

## Conclusions

The presence of a granulocytic sarcoma found within the GI tract in patients with myelodysplastic syndrome is a significantly rare occurrence. This case report is presented with hopes to provide an index of suspicion given past medical history of myelodysplastic syndrome with signs and symptoms of bowel obstruction. Given the complicated nature of this disease, patients require a multidisciplinary care approach to ensure improved outcomes. Medical therapy should be a primary management modality, but in cases of complicated disease, such as bowel obstruction, surgical intervention should be pursued.
